# Umbilical Coiling Index as a Marker of Perinatal Outcome: An Analytical Study

**DOI:** 10.1155/2012/213689

**Published:** 2012-02-14

**Authors:** T. Chitra, Y. S. Sushanth, S. Raghavan

**Affiliations:** Department of Obstetrics and Gynecology, Jawaharlal Institute of Postgraduate Medical Education and Research, Puducherry, No.3, E-Type Quarters, JIPMER Campus, India

## Abstract

*Objectives*. To measure umbilical coiling index (UCI) postnatally and to study the association of normocoiling, hypocoiling and hypercoiling to maternal and perinatal outcome. *Method(s)*. One thousand antenatal women who went into labour were studied and umbilical coiling index calculated at the time of delivery. UCI was determined by dividing the total number of coils by the total umbilical cord length in centimeters. Its association with various maternal and perinatal risk factors were noted. The statistical tests were the Chi-square test and assessed with SPSS version 13.0 software and statistically analyzed. *P* value of less than 0.05 was regarded as statistically significant. *Results*. The mean umbilical coiling index was found to be 0.24 ± 0.09. Hypocoiling (<0.12) was found to be significantly associated with hypertensive disorders, abruptio placentae, preterm labour, oligohydramnios, and fetal heart rate abnormalities. Hypercoiling (>0.36) was found to be associated with diabetes mellitus, polyhydramnios, cesarean delivery, congenital anomalies, and respiratory distress of the newborn. *Conclusion*. Abnormal umbilical coiling index is associated with several antenatal and perinatal adverse features.

## 1. Introduction

 Umbilical cord is vital to the development, well-being, and survival of the fetus, yet this is vulnerable to kinking, compressions, traction, and torsion which may affect the perinatal outcome. The umbilical cord is protected by Wharton's jelly, amniotic fluid, helical patterns, and coiling of vessels. The origin of umbilical cord coiling is unknown. Hypotheses include fetal movements, active or passive torsion of the embryo, differential umbilical vascular growth rates, fetal hemodynamic forces, and the arrangements of muscular fibers in the umbilical arterial wall. Of the many characteristics of the human umbilical cord, a most mysterious and intriguing one is the twisted or spiral course of its component blood vessels. Mathematically speaking, the vessels of the cord are wound as cylindrical helices, rather than spirals, but both terms are used interchangeably to avoid confusion [1]. The coiling of the umbilical vessels develops as early as 28 days after conception and is present in about 95% of fetuses by 9 weeks of conception. The helices may be seen by ultrasonographic examination as early as during the first trimester of pregnancy [2].

The spiral course of the umbilical vessels was first recorded by Berengarius in 1521. It was then confirmed by Columbus in 1559 and by Arantius in 1564. In 1600, Fabricius demonstrated that both right (dextral) and left (sinistral) helices of the umbilical cord exists [3]. If umbilical cord twists were to be determined randomly, one would expect both forms of twists to be equal in incidence. However, many investigators have found that majority of the cords have a left-sided twist [1, 2, 4].

The number of twists seen in first trimester is roughly the same as that seen in term cords. The total number of coils seen is between 0 and 40. Umbilical coiling appears to confer turgor to the umbilical unit, producing a cord that is strong, yet flexible. Since lengthening of the cord occurs from the fetal end, perhaps coiling of the cord represents a long-term record of fetal well-being [4]. 

A coil is of 360-degree spiral course of umbilical vessels. Umbilical cord index (UCI) is defined as the total number of coils divided by the total length of the cord in centimeters. A frequency distribution of umbilical cord index (UCI) was done by Rana et al. (1995) [5].

They grouped the UCI as follows:

<10th percentile—hypocoiled;
10th–90th percentile—normocoiled;
>90th percentile—hypercoiled.

The difference in coiling was described as an antenatal marker identifying fetus at risk. Majority of the studies on UCI have been done postnatally. Although UCI can be calculated antenatally by ultrasonography, limited data is available as to its accuracy. Our present study aims at calculating the UCI postnatally and identifying the association between perinatal morbidity and mortality. 

## 2. Materials and Methods

A prospective analytical study was performed in our institute over a period of 2 years. This study was approved by institute ethical committee. A total number of one thousand pregnant women were randomly chosen by a single observer from those who got admitted to the labour ward. Only deliveries after 28 weeks with singleton, cephalic presentations were included in the study. Multiple pregnancies, malpresentations, previously diagnosed IUD, and elective caesarean section were excluded. The last two factors were excluded to avoid bias during computation of statistics. 

Immediately after delivery, the umbilical cord was clamped at the fetal end and cut with scissors taking care not to milk the cord (as the latter might affect the UCI). The placenta was allowed to separate spontaneously. Any significant postpartum events like postpartum hemorrhage (PPH), genital tract injuries, inversion, or postpartum collapse were noted. At the fetal end, the cord was cut 5 cm from the fetal insertion. The rest of the cord from the cut end to the placental insertion was measured (in centimeters). No excessive traction was exerted on the cord at the time of measurement. Five cms was added to the length of the measured cord. A coil was taken as one complete 360-degree spiral course of the umbilical vessels.

The number of coils of the entire cord was counted:

(1) $$\begin{array}{cc} & \text{umbilical}\;\text{coiling}\;\text{index}\\ & \;\;=\frac{\text{total}\;\text{number}\;\text{of}\;\text{complete}\;\text{vascular}\;\text{coiling}}{\text{Total}\;\text{length}\;\text{of}\;\text{cord}\;\text{in}\;\text{cms}}. \end{array}$$

The following maternal factors were recorded age, parity, anemia, pregnancy induced hypertension (PIH), blood group, heart disease, infertility, gestational diabetes mellitus (GDM), gestational age, premature rupture of membranes (PROM), placenta praevia, abruption placentae, chorioamnionitis, oligohydramnios, and polyhydramnios. Intrapartum factors like mode of delivery, fetal heart rate (FHR) abnormalities, meconium stained liquor (MSL), and postpartum hemorrhage (PPH) were noted. Neonatal factors like APGAR, birth weight, admission to neonatal intensive care unit (NICU), and congenital anomaly were also noted. At the end of sample collection, the mean UCI was calculated. On the basis of the latter, they were grouped as *normocoiled* group having UCI values between the 10th and 90th percentile of the mean UCI. *Hypocoiled* group was taken as having values less than the 10th percentile and *hypercoiled* group and having values more than 90th percentile of the mean.

The hypocoiled and hypercoiled groups were compared with the normocoiled group, and associations of the chosen parameters with UCI were studied. The statistical tests were the Chi-Square test and the Fisher's exact test. The values were entered and assessed with SPSS version 13.0 software and statistically analyzed. *P* value of less than 0.05 was regarded as statistically significant.

## 3. Results

The mean length of the umbilical cord was found to be 52.87 + 13.49 cms. The mean number of coils per umbilical cord was found to be 12.59 + 5.38. The mean UCI was 0.24 + 0.09 coils per cms (Table 1). Normocoiled were predominant in 78.3%, 11.7% cases were hypocoiled, and 10.0% were hypercoiled.

The distribution frequencies of the three groups according to the maternal factors have been shown in Tables 2 and 3.

Of the antenatal risk factors studies, age more than 35 years was found to have a significant association with hypocoiling (*P* = 0.041) and hypercoiling (*P* = 0.003). Hypertensive disorders were found to be significantly associated with hypocoiling (*P* = 0.030). Diabetes mellitus had a statistically significant association with hypercoiling (*P* = 0.035) (Table 2). There was no association found between UCI and risk factors such as parity, anaemia, Rh status, heart disease, and infertility.

Abruptio placenta had a significant association with hypocoiling (*P* = 0.019). Preterm labour and oligohydramnios were found to be significantly associated with hypocoiling (*P* = 0.004  and 0.013), whereas polyhydramnios was found to be significantly associated with hypercoiling (*P* = 0.015). No significant association was found between UCI and PROM, prolonged pregnancy, placentas praevia, epilepsy, and chorioamnionitis (Table 3).

FHR abnormalities {prolonged decelerations (17.6%), tachycardia (0.5%)} were associated with both hypocoiling (18.8%) and hypercoiling (16.6%) which was highly significant (*P* < 0.001). Both hypocoiled and hypercoiled were significantly associated with MSL (*P* = 0.020, *P* < 0.001). PPH was also found to be significantly associated with hypercoiling (*P* = 0.006). There was no significant association found between instrumental deliveries (17.2%) and umbilical coiling, but hypercoiling was significantly associated with caesarean section (*P* = 0.001) (Table 4).

The mean APGAR was found to be 7.70, 8.73, and 8.79 at 1, 5 and 10 minutes, respectively. Both hypocoiling and hypercoiling were found to be statistically significant associated with perinatal hypoxia (*P* = 0.047, *P* = 0.014), low-birthweight (LBW) babies (*P* = 0.011), and hypercoiling was highly significantly associated with congenital anomalies (*P* < 0.001) (Table 5). Both hypocoiling and hypercoiling were found to be significantly associated with prematurity (*P* = 0.023, *P* = 0.018) and respiratory distress (*P* = 0.005, *P* = 0.009). There was no significant association noted between meconium aspiration syndrome and abnormal umbilical coiling. 

## 4. Discussion

The umbilical coiling index has been found to be an effective indicator of perinatal outcome. The aim of this study was to find the relationship between UCI and various maternal and perinatal factors. The mean UCI in our study was 0.24 + 0.09 which was similar to the study done by Ezimokhai et al. (2001) (Table 6) [6].

Among the antenatal risk factors, our study found an association between elderly gravida (>35 years) and both hypocoiled and hypercoiled (*P* = 0.041 and *P* = 0.003, resp.). Among previous studies, Ezimokhai et al. [9] found hypercoiled to be associated with extremes of maternal age (<20 and >35 years). None of the other studies found age to be a significant factor. Our study did not find any significant association with parity, anemia, Rh negative pregnancy, presence of heart disease, or infertility. No significant association was found between UCI and any of these factors in previous studies also.

Preeclampsia was found to have a significant association with hypocoiled (*P* = 030). Ezimokhai et al. [9] also demonstrated a significant association between noncoiled (an extreme form of hypocoiled) cords and preeclampsia. Similar findings were found in studies done by Gupta et al. [10]. The coiled umbilical cord, because of its elastic properties, is able to resist external forces that might compromise the umbilical vascular flow. The coiled umbilical cord acts like a semierectile organ that is more resistant to snarling torsion, stretch, and compression than the noncoiled one [10, 11]. This might explain the association of hypocoiling with preeclampsia. In our study, diabetes mellitus was found to be significantly associated with hypercoiled (*P* = 0.035). Ezimokhai et al. [6, 9], however, found significant association of GDM with both hypocoiled and hypercoiled.

Abruptio placentae was found to have a significant association with hypocoiled (*P* = 0.019). No studies have demonstrated this association so far. Probably, the high association of preeclampsia with abruption contributed to this finding.

Machin et al. [11] and de Laat et al. [12] found a significant association between hypocoiled and chorioamnionitis. But our study failed to demonstrate any significant association between chorioamnionitis and UCI (*P* = 0.361). The fact that cases of chorioamnionitis were found in only 1% of the study sample might be the reason for our observation. Kashanian et al. [13] found oligohydramnios to be significantly associated with both hypocoiled and hypercoiled. In our study, oligohydramnios had a significant association with hypocoiled (*P* = 0.013), whereas polyhydramnios had a significant association with hypercoiled (*P* = 0.015). This can be explained by Edmond's hypothesis [3] which states that twist of the umbilical cord is a result of the rotary movement imparted to the embryo, and hence more is the liquor amnii, more is the rotary movement of the fetus and more will be the coiling. The converse will be true for oligohydramnios.

Our study found a significant association between preterm labour and hypocoiled (*P* = 0.004). This was consistent with the findings of Strong et al. [4] and de Laat et al. [8]. Both these authors, however, were unable to give a satisfactory explanation. Rana et al. [5] and de Laat et al. [12] found hypercoiled to be significantly associated with preterm labour. They believed that hypercoiled was an adaptive response to fetal hemodynamic changes, which initiates preterm labour on reaching a certain threshold. 

FHR variations were found to have a highly significant association with both hypocoiled and hypercoiled. In both instances, *P* value was less than 0.001. Literature has found a consistent association between intrapartum FHR decelerations and abnormal UCI. Strong et al. [4] and de Laat el al. [14] found FHR decelerations to be associated with both hypocoiled and hypercoiled. According to them, hypocoiled and hypercoiled cords are less flexible or more prone to kinking and torsion which makes them less tolerant to withstand the stress of labour. Rana et al. [5] and Ercal et al. [7] found FHR decelerations to be significantly associated with hypocoiled. Rana et al. [5] felt that coiling provides turgor and compression resistant properties to the cord which become compromised as the cord becomes hypocoiled. 

Meconium staining of the amniotic fluid was found to have a significant association with both hypocoiled (*P* = 0.020) and hypercoiled (*P* < 0.001). Although Similar findings were noted in studies done by Strong et al. [4, 15] and Ezimokhai et al. [9], they did not offer a specific explanation for the observation.

Instrumental deliveries did not have any association with extremes of UCI in our study. Caesarean deliveries, however, were found to have highly significant association with hypercoiled (*P* = 0.001). Many authors have found a positive association between operative delivery, especially for fetal distress and abnormal UCI [4, 5, 14]. In our study, caesarean sections done for all obstetric indications were included and not just those which were done only for fetal distress. This may explain as to why operative delivery was associated with hypercoiled, whereas other authors found an association with hypocoiled [4, 5, 14, 15]. 

PPH, in our study, was found to have a significant association with hypercoiled (*P* = 0.006). This relationship has not been demonstrated in any of the previous studies. Probably, the association of PPH with factors like multiple pregnancy and polyhydramnios, which are related to hypercoiled, may result in a linear relation.

An initial low APGAR (<6 at 5 minutes) was found to have a significant relationship with both hypocoiled and hypercoiled in our study. The *P* values were 0.047 and 0.014, respectively. A similar result was obtained by Gupta et al. [10] and Kashanian et al. [13]. When the birth weight of babies were compared with UCI, it was found that LBW (birth weight <2.5 kg) was significantly associated with both hypocoiled (*P* = 0.011) and hypercoiled (*P* = 0.001). Literature has found a consistent association between hypercoiled and LBW babies, as shown by Rana et al. [5], Raio et al. [16], and de Laat et al. [12]. However, the authors were unable to give a satisfactory explanation for this casual association.

Respiratory distress was found to be significantly associated with both hypocoiled (*P* = 0.005) and hypercoiled (*P* = 0.009) in our study. De Laat et al. [8, 12] found hypercoiled to be significantly associated with birth asphyxia-acute and chronic. Again, the reason may be derived linearly from the associations between FHR decelerations, operative delivery, and initial low APGAR.

Our study demonstrated a significant association between IUGR babies and hypocoiled (*P* = 0.004). Strong et al. [4] and Machin et al. [11] obtained a similar result in their studies. They summarized that since adequate coiling prevents compression of the cord, hypocoiling in the long run, results in reduced fetoplacental circulation, thus resulting in growth restriction. Ezimokhai et al. [9] and de Laat et al. [14] found IUGR to be associated with hypercoiled.

According to the former, hypercoiled predisposes to more kinking and torsion of the cord, again interfering in fetoplacental circulation. Congenital anomalies were found to be significantly associated with hypercoiled (*P* < 0.001). Strong et al. [4] and Ezimokhai et al. [9], however, found anomalies to be significantly associated with hypocoiled, although a satisfactory explanation was not forthcoming.

To conclude, abnormal umbilical coiling index is associated with several adverse antenatal and neonatal features. The association shows wide variations among the various studies done so far. Antenatal study of UCI should be further pursued to confirm diagnosis at an earlier gestational age.

## Figures and Tables

**Table 1:** Umbilical coil characteristics.

	*N *	Minimum	Maximum	Mean	Standard error	Standard deviation
Length (cm)	1000	20	110	52.87	0.43	13.49
No. of coils	1000	2	45	12.59	0.18	5.38
UCI	1000	0.06	0.62	0.24	0.003	0.09

**Table 2:** Distribution frequencies of the three groups according to the maternal factors.

	20–34 years	>35 years	*P* value
Hypocoiled	99	5	0.041*
Normocoiled	702	11	
Hypercoiled	90	5	0.003**

UCI normal	(*N* = 857)	PIH (*N* = 143)	*P* value

Hypocoiled	93 (10.9%)	24 (16.8%)	0.030
Normocoiled	681 (79.5%)	102 (71.3%)	
Hypercoiled	83 (9.7%)	17 (11.9%)	0.273

UCI normal	(*N* = 990)	Diabetic (*N* = 10)	*P* value

Hypocoiled	114 (11.5%)	3 (30%)	0.051
Normocoiled	779 (78.7%)	4 (40%)	
Hypercoiled	97 (9.8%)	3 (30%)	0.035*

**Table 3:** Distribution frequencies of the three groups according to the maternal factors.

	Normal (*N* = 901)	Preterm (*N* = 99)	*P* value
Hypocoiled	97 (10.8%)	20 (20.2%)	0.004*
Normocoiled	718 (79.7%)	65 (65.7)	
Hypercoiled	86 (9.5%)	14 (14.1%)	0.060

UCI	Normal (*N* = 987)	Abruption (*N* = 13)	*P* value

Hypocoiled	112 (11.3%)	5 (38.5%)	0.019*
Normocoiled	775 (78.5%)	8 (61.5%)	
Hypercoiled	100 (10.1%)	0 (0%)	0.310

UCI	Normal (*N* = 950)	Oligohydramnios (*N* = 50)	*P* value

Hypocoiled	105 (11.1%)	12 (24.0%)	0.013*
Normocoiled	749 (78.8%)	34 (68.0%)	
Hypercoiled	96 (10.1%)	5 (27.8%)	0.015*

UCI	Normal (*N* = 950)	Polyhydramnios(*N* = 50)	*P* value

Hypocoiled	113 (11.5%)	4 (22.2%)	0.076
Normocoiled	774 (78.8%)	9 (50.0%)	
Hypercoiled	95 (9.7%)	5 (27.8%)	0.015*

**Table 4:** Distribution frequencies of the three groups according to the intrapartum factors.

UCI	Spontaneous vaginal delivery (*N* = 735)	Caesarean section (*N* = 93)	*P* value
Hypocoiled	78 (10.6%)	15 (16.1%)	0.060
Normocoiled	595 (81.0%)	61 (65.6%)	
Hypercoiled	62 (8.4%)	17 (18.3%)	0.001*

UCI	Spontaneous vaginal delivery (*N* = 735)	Instrumental deliveries (*N* = 172)	

Hypocoiled	78 (10.6%)	24 (14.0%)	0.188
Normocoiled	595 (81.0%)	127 (73.8%)	
Hypercoiled	62 (8.4%)	21 (12.2%)	0.110

UCI	Normal (*N* = 819)	Abnormal FHR (*N* = 181)	*P* value

Hypocoiled	83 (10.1%)	34 (18.8%)	<0.001*
Normocoiled	666 (81.3%)	117 (64.6%)	
Hypercoiled	70 (8.5%)	30 (16.6%)	<0.001*

UCI	Normal (*N* = 800)	MSL (*N* = 200)	*P* value

Hypocoiled	86 (10.8%)	31 (15.5%)	0.020*
Normocoiled	649 (81.1%)	134 (67.0%)	
Hypercoiled	65 (8.1%)	30 (16.6%)	<0.001*

UCI	Normal (*N* = 963)	PPH (*N* = 37)	*P* value

Hypocoiled	112 (11.6%)	5 (13.5%)	0.396
Normocoiled	760 (78.9%)	23 (62.2%)	
Hypercoiled	91 (9.4%)	9 (24.3%)	0.006*

**Table 5:** Distribution frequencies of the three groups according to the neonatal factors.

	Normal (*N* = 949)	Low APGAR (*N* = 51)	*P* value
Hypocoiled	107 (11.3%)	10 (19.6%)	0.047*
Normocoiled	752 (79.2%)	31 (60.8%)	
Hypercoiled	90 (9.5%)	10 (19.6%)	0.014*

UCI	Normal (*N* = 723)	LBW (*N* = 238)	*P* value

Hypocoiled	74 (10.2%)	37 (15.5%)	0.011*
Normocoiled	589 (81.5%)	165 (69.3%)	
Hypercoiled	60 (8.3%)	36 (15.61)	0.001*

UCI	Normal (*N* = 978)	Anomalies (*N* = 22)	*P* value

Hypocoiled	113 (11.6%)	4 (18.2%)	0.186
Normocoiled	773 (79.0%)	10 (44.5%)	
Hypercoiled	92 (9.4%)	8 (37.3%)	<0.001*

**Table 6:** Mean UCI in various studies.

	UCI
Strong et al. [4]	0.21 + 0.07
Rana et al. [5]	0.19 + 0.1
Ercal et al. [7]	0.20 + 0.1
Ezimokhai et al. [6, 9]	0.26 + 0.09
de Laat et al. [8]	0.17 + 0.009
Present study	0.24 + 0.09
